# Twin vocal folds as a novel evolutionary adaptation for vocal communications in lemurs

**DOI:** 10.1038/s41598-024-54172-z

**Published:** 2024-02-13

**Authors:** Kanta Nakamura, Mayuka Kanaya, Daisuke Matsushima, Jacob C. Dunn, Hideki Hirabayashi, Kiminori Sato, Isao T. Tokuda, Takeshi Nishimura

**Affiliations:** 1 Center for the Evolutionary Origins of Human Behavior, Kyoto University, Inuyama, Aichi Japan; 2 College of Science and Engineering, Ritsumeikan University, Kusatsu, Shiga Japan; 3 Behavioural Ecology Research Group, Anglia Ruskin University, Cambridge, UK; 4 Biological Anthropology, The University of Cambridge, Cambridge, UK; 5 Department of Cognitive Biology, University of Vienna, Vienna, Austria; 6 Dokkyo Medical University, Mibu, Tochigi Japan; 7 Department of Otolaryngology-Head and Neck Surgery, Kurume University School of Medicine, Kurume, Fukuoka Japan

**Keywords:** Biological anthropology, Biomechanics

## Abstract

Primates have varied vocal repertoires to communicate with conspecifics and sometimes other species. The larynx has a central role in vocal source generation, where a pair of vocal folds vibrates to modify the air flow. Here, we show that Madagascan lemurs have a unique additional pair of folds in the vestibular region, parallel to the vocal folds. The additional fold has a rigid body of a vocal muscle branch and it is covered by a stratified squamous epithelium, equal to those of the vocal fold. Such anatomical features support the hypothesis that it also vibrates in a manner like the vibrations that occur in the vocal folds. To examine the acoustic function of the two pairs of folds, we made a silicone compound model to demonstrate that they can simultaneously vibrate to lower the fundamental frequency and increase vocal efficiency. Similar acoustic effects are achieved using different features of the larynx for the other primates, e.g., by vibrating multiple sets of ventricular folds in several species and further by an evolutionary modification of enlarged larynx in howler monkeys. Our multidisciplinary approaches found that these functions were acquired through a unique evolutionary adaptation of the twin vocal folds in Madagascan lemurs.

## Introduction

Non-human primates exhibit a range of different call types in their vocal repertoires^1^. Call production is largely based on the same acoustic and physiological principles as speech production in humans^2–5^. First, the vocal source is generated by vibrations of the vocal fold/membrane complex in the larynx^6–8^. This is then modulated by resonance in the vocal tract filter to emit a communicative signal with amplified formants^9,10^. Phonetic information, such as loudness, pitch, and duration, is primarily determined by the acoustic properties of the vocal source. Thus, the vocal repertoire is principally determined by the process of laryngeal source generation in non-human primates^4–7^.

Recent studies have provided empirical evidence for several phonatory mechanisms to vary the acoustic structure of the vocal source in non-human primates. Non-human primates have a vocal membrane, a superior extension from the vocal fold, to efficiently generate a diverse set of complex vocal signals without the need for correspondingly complex motor control^6,7^. Additional vibrations of laryngeal vestibular folds (also termed ventricular folds) are used to lower the pitch of calls in macaques^11^, elephants, pigs, and bats^12–14^. Specializations in laryngeal anatomy have also been documented in several primate species. For example, howler monkeys (*Alouatta* spp.) are well known to have a grossly enlarged larynx, with a huge bullate hyoid bone into which a laryngeal air sac extends^15–17^. Many other species are also known to possess varied forms of air sacs, which likely serve to amplify calls and/or exaggerate body size^15,18,19^. Such physiological and anatomical variation of the laryngeal region is likely to impact the vocal source acoustics, thereby diversifying call repertoire.

Lemuriform primates have experienced isolated evolutionary diversification on Madagascar since their divergence from Afro-Eurasian lorisiforms 50–60 million years ago^20–22^. Several anatomical specializations of the larynx have been described for this clade. For example, in ring-tailed lemurs (*Lemur catta*), a valve-like structure other than the vocal fold has been described^19^, and the thyro-arytenoid (TA) muscle (or vocal muscle) is known to extend into the laryngeal vestibule as well as into the vocal folds (VFs)^15^. In both ring-tailed lemurs and brown lemurs (*Eulemur mongoz*) a deep sulcus between the vestibule and epiglottis has also been described^4,18^. Air sacs are not thought to be present in these species^4,15,18^, but an air sac extends dorsally from the larynx in black-and-white ruffed lemurs (*Varecia variegate*)^15,19^. While these anatomical features have thus far only been described anatomically, rather than functionally, we would predict that such specializations are related to vocal source acoustics in these species^23–26^.

Here, we document newly described adaptations to the gross- and histo-anatomy of the larynx in lemuriforms and provide empirical evidence of their acoustic contributions with model simulation experiments. This multidisciplinary approach offers further insight into how the evolutionary flexibility of the larynx has led to the diversification of vocal repertoires in non-human primates^27^.

## Results and discussion

**Figure 1:**
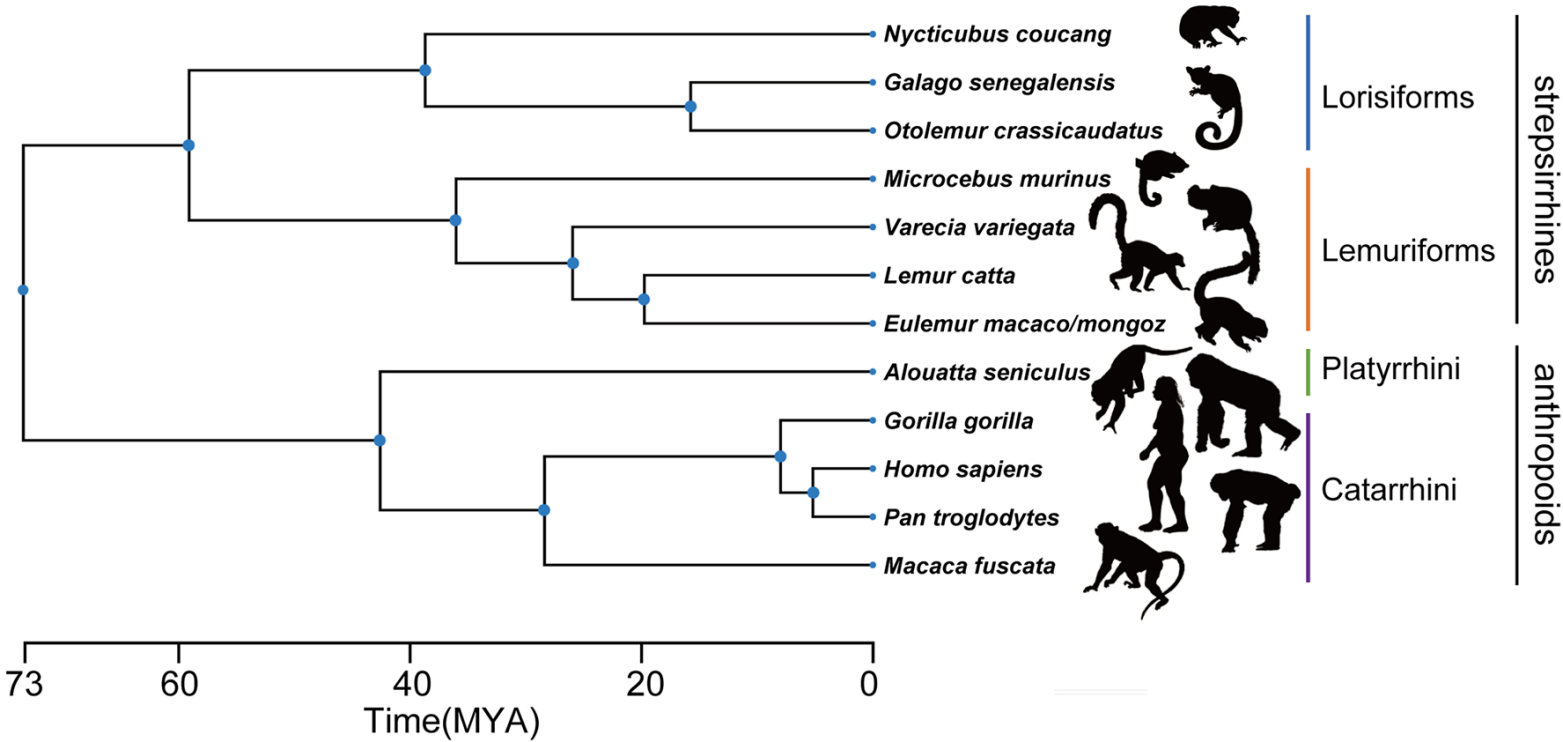
Phylogenetic relationships among the primate species examined and described in this study. Topology and divergence times from TimeTree^28^—horizontal axis shows divergence times in million years before present. Silhouettes from PhyloPic (http://phylopic.org/).

The larynges from five species/four genera/two families of lemuriforms, and three species/three genera/two families of lorisiforms^20–22^ were examined with micro-computed tomography (μCT) and compared with those of anthropoid primates from the literature, including humans as an outgroup^4,6,15,18^ (Fig. 1 and Supplementary Table 1).

**Figure 2:**
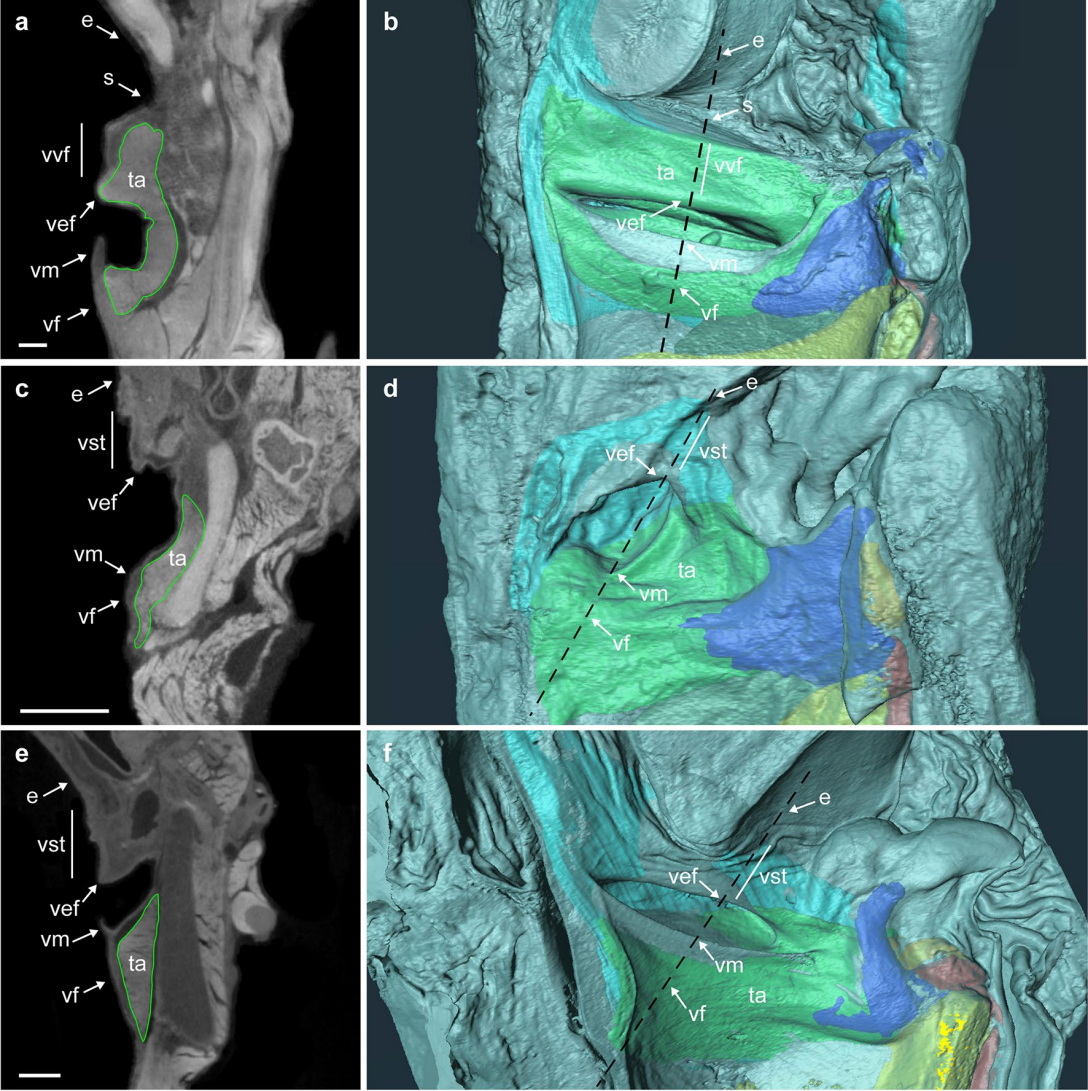
Gross-anatomy of the larynx. (**a**, **b**) CT frontal scans and 3D images of *Lemur catta* (lemuriform); (**c**, **d**) *Nycticebus coucang* (lorisiform); and (**e**, **f**) *Macaca fuscata* (anthropoid). Scale 1 mm. The cutting planes of (**a**), (**c**), and (**e**) are the dashed lines on (**b**), (**d**), and (**f**), respectively. e, epiglottis; s, sulcus between the vestibule and the epiglottis; ta (green), thyro-arytenoid muscle; vef, vestibular fold; vf, vocal fold; vm, vocal membrane; vst, vestibule; and vvf, vestibular vocal fold. dark blue, arytenoid cartilage; yellow, cricoid cartilage; red, posterior cricoarytenoid muscle; and light blue, thyroid cartilage.

All species of lemuriforms and lorisiforms studied were found to have a laryngeal ventricle to separate the vestibule from the VF and vocal membrane (Fig. 2a–d and Supplementary Figs. 1 and 2). TA muscle forms the body of the VF, connecting the thyroid to the arytenoid cartilages (Fig. 2a–d and Supplementary Figs. 1 and 2a–c). The thick lamina propria covers the TA muscle (Fig. 2a, c and Supplementary Fig. 1). Such features are similar to those seen in anthropoids^6,15^ (here, the larynges from a macaque, gorilla, and chimpanzee were examined as references; Fig. 2e, f and Supplementary Fig. 2d).

All lemuriforms examined here, regardless of sex, had a deep and long sulcus between the vestibule and epiglottis, forming a thick fold in the vestibule (vvf, Fig. 2a, b, Supplementary Figs. 1a–d, 2a, b, and 3a–c, 4). The fold in the vestibule is parallel to the VF (Fig. 2b, Supplementary Fig. 2a, b and 3a–c, 4). Our μCT scans showed that the fold in the vestibule comprises a branch of TA muscle covered by the thick lamina propria (Fig. 2a, b, Supplementary Figs. 1a–d, 2a, b, and 3a–c). The anatomical features indicate that when the arytenoid cartilage is adducted, the rigid body of TA muscle drives the vestibule’s folds to move to the midline of the laryngeal cavity, in a similar way to the VFs. Interestingly, black-and-white ruffed lemurs have a thin membrane-like extension protruding from this fold, which appears to be very similar to the vocal membrane of the VF (Supplementary Figs. 1c and 2c). Thus, the fold found in the vestibule is comparable with the VF in anatomical terms. We propose the term ‘vestibular vocal fold’ (VVF), for this novel laryngeal feature, which differs from the vestibular fold formed at the lower edge of the vestibule.

The lorisiforms examined here show no or a quite shallow sulcus between the vestibule and epiglottis, forming a tiny fold at the upper edge of the vestibule (vst, Fig. 2c, d, Supplementary Figs. 1e, f, 2c and 3d–f). The fold has no branch of TA muscle (Fig. 2c, d, Supplementary Figs. 1e, f, and 2c). In anthropoids, no sulcus is developed, and no branch of TA muscle is found within the vestibule^4,6,15,18^ (vst, Fig. 2e, f, Supplementary Figs. 2d, and 3g). The absence of the TA muscle likely precludes an active and effortless air space closure in the vestibular region in the lorisiforms and anthropoids. Thus, the VVF, with a rigid body of TA muscle, is likely a derived evolutionary feature in a clade of lemuriforms.

**Figure 3:**
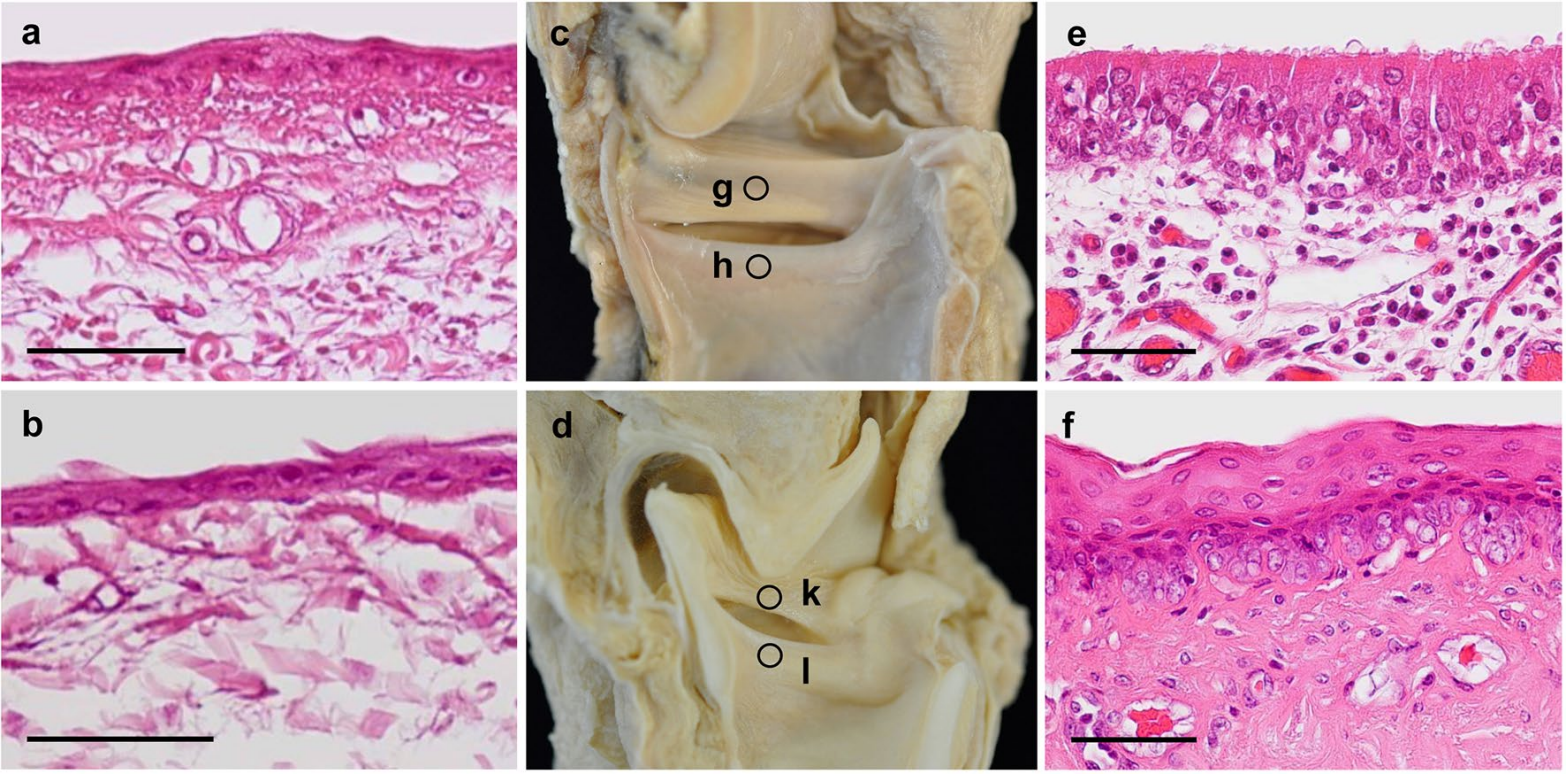
Histo-anatomy of the larynx. (**a**–**c**) H & E stained sections with a medial view of larynges of *Lemur catta* and d–f *Macaca fuscata*: (**a**, **e**) vestibule and (**b**, **f**) vocal fold, (**c**, **d**) a medial view. The sections of (**a**/**b**) and (**e**/**f**) were made at the circles on (**c**) and (**d**), respectively. Scale 50 μm.

We also found that the VVF is comparable with the VF in histo-anatomical terms. The sections stained with hematoxylin and eosin (H & E) in a ring-tailed lemur showed that the VVF is covered with two- or three-layered stratified squamous epithelium, as found in the VF (Fig. 3a–c). In humans, only the VF is covered with this type of epithelium, but other laryngeal regions, including the vestibule, are covered with the pseudostratified ciliated epithelium^29,30^. The stratified squamous epithelium has a high tensile strength and is flexible, thus becoming quite resistant to mechanical stimuli and friction^29,30^. This feature reflects the frequent collisions of the VFs in humans. Similar gross and histo-anatomy was found in other anthropoids, which do not possess VVFs (Fig. 3d–f and Supplementary Fig. 5). The glottal collisions by the VFs cause abrupt cessations of the transglottal airflow during vocalization, and play a key role in vocal source generation by adding higher harmonics indispensable for producing varied voiced calls^31^. Vibrations without collisions, on the other hand, play only a minor role in vocal production^31^. Our findings suggest that uniquely in lemuriforms frequent collisions also occur in a pair of VVFs, similar to the action of VFs in other clades.

**Figure 4:**
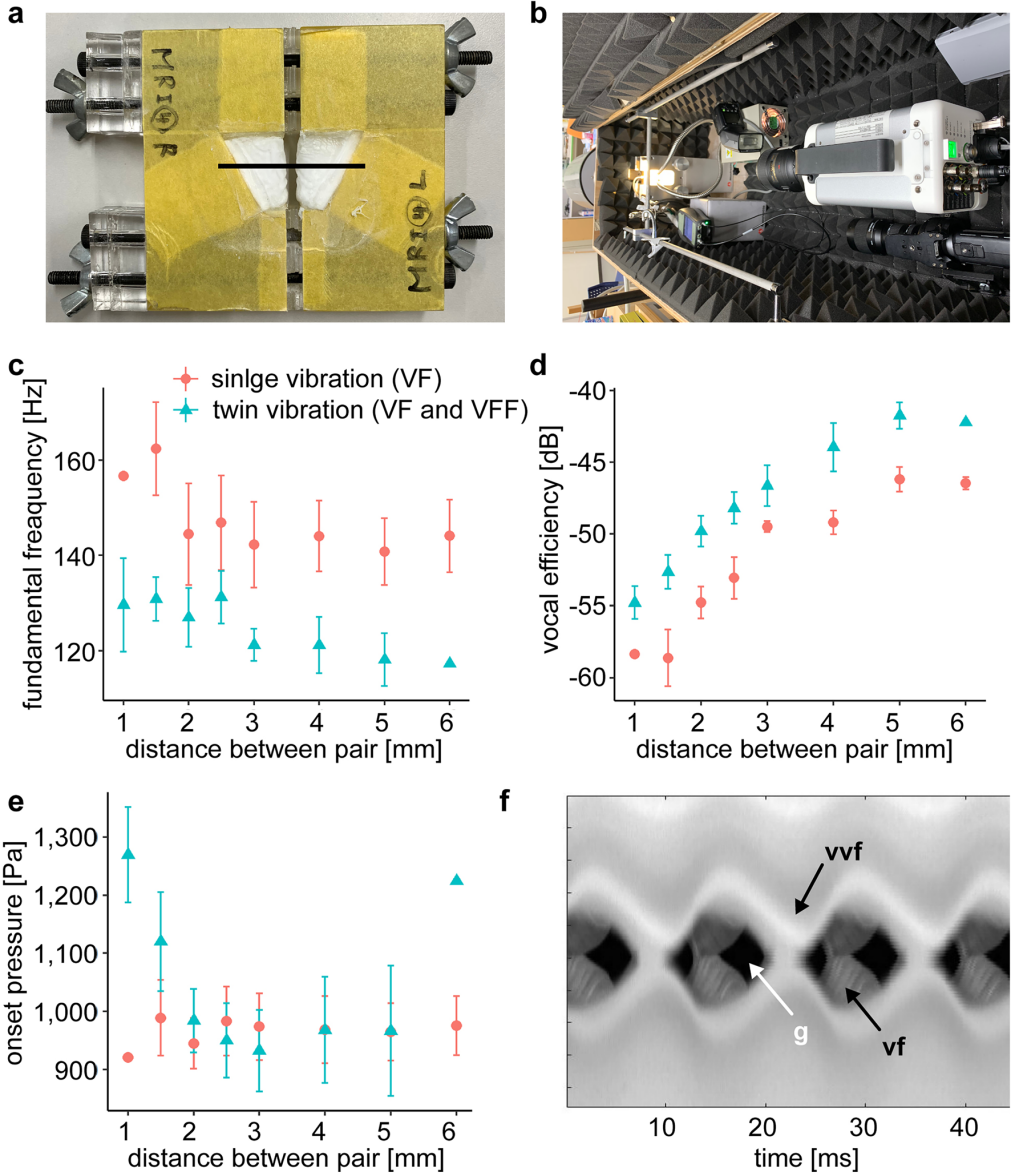
Measurements and kymogram of the silicone compound model experiment. (**a**) Fabricated silicone model (representing VFs and VVFs). (**b**) Experimental equipment of high-speed camera (right) and models (left). Plots showing: (**c**) Fundamental frequency. (**d**) Vocal efficiency. (**e**) Onset pressure. The error bars represent the standard error of the data measured. (**f**) Kymogram of the twin model with a distance of 2.5 mm (flow rate: 1.7 l/min, subglottal pressure: 1.6 kPa). Recorded vibration on the black thick line in (**b**). g, glottal opening; vf, VF model; and vvf, VVF model.

Our gross- and histo-anatomical findings strongly suggest that the lemuriforms use the twin vocal folds—the VFs and VVFs—to produce vocalizations. Because in vivo and ex vivo evidence of phonation is scarce in lemurs for methodological reasons, to examine the acoustic function of the twin vocal folds, we utilized a silicone compound model developed to study human VF vibration (Fig. 4a, b)^32,33^. Such models have proven extremely useful for understanding the physiology of vocal production, since their oscillation properties have been deeply investigated and the essential features of animal vocalizations can be captured by such models^2,3,6,8,12,34^. The vocal membrane and membrane-like extensions were not included in the VF models used here, since this would drastically increase model complexity, through their mechanical and aerodynamic interactions with the VF. Here, we aim to explore dynamic relationship between the VFs and VVFs, and thus we used simplified models without membrane-like features.

Airflow was injected through two pairs of human VF models, where the upper pair (representing the VVFs) was located downstream of the lower pair (representing the VFs, Fig. 4b). This produced flow-induced oscillations of the twin pairs of folds. To increase intraglottal pressure, the VVFs were adducted such that their opening was narrowed. As the main control parameter to adjust the adduction level of the VVFs, the medial–lateral distance between the left and right VVFs was changed. To highlight the effect of the VVFs, the same experiment was carried out only for the oscillation of the VFs without the oscillation of the VVFs.

A comparison of the experiments with and without oscillation of VVFs revealed that co-oscillation of the VFs and VVFs significantly lowered the fundamental frequency and highly improved vocal efficiency (fundamental frequency: *p* = 3.927 × 10^–7^, distance *p* = 1.238 × 10^–3^, interaction *p* = 0.961; vocal efficiency: *p* = 1.542 × 10^–4^, distance *p* = 2.455 × 10^–7^, interaction *p* = 0.921, Fig. 4c, d), but had only a minor influence on the onset pressure, compared with when only the VFs oscillated (*p* = 0.118, distance *p* = 0.932, interaction *p* = 0.713, Fig. 4e). Next, we examined oscillation patterns of the VFs and VVFs using a kymogram that visualizes medial–lateral movements of the edges of the two pairs of folds^35,36^. Although the varied distance between the left and right VVFs induced a slight change in the oscillation pattern, the VVFs always vibrated out-of-phase with the VFs (Fig. 4f). Such out-of-phase oscillations of two interacting systems can extend the oscillation period, compared with that of a single system, by accommodating two peaks (or troughs) within one oscillation cycle. This could explain why the fundamental frequency was lowered in the co-oscillations of the VFs and VVFs. Thus, the VVFs likely vibrate to lower the fundamental frequency and increase vocal efficiency in lemuriform vocalizations.

Our results suggest that lemuriforms acquired the twin vocal folds as a novel evolutionary adaptation. We examined the larynges from two families of lemuriforms, including Lemuridae and Cheirogaleidae, both of which show VVFs. Thus, this derived feature was probably acquired at the latest by their common ancestor about 35 million years ago^21^ (Fig. 1).

The simultaneous vibrations of the twin pairs of vocal folds probably improve voice efficiency and lower fundamental frequency. Such acoustic adaptation may facilitate the production of loud and/or lower-frequency calls. Our anatomical findings suggest that the contraction status of the TA muscles determine whether there was a switch between the vibrations of single or twin pairs of oscillators, even during a single call. A loud long-distance call is often observed in lemuriforms^23–26^, and the twin vocal folds may be involved in the efficient production of such calls in the repertoire. Although not examined here, the membrane-like extension from the VVFs would increase their complexity in the mechanical and aerodynamic interactions with the main body of the VVFs, e.g., deterministic chaos^6^. Thus, the twin vocal folds, and their membrane like extensions found in some species, probably increase acoustic complexity and allow for variation in vocal production within and among species’ repertoires in the Madagascan primates.

Specializations of the hyo-laryngeal apparatus has occurred independently in several primate clades^15,16^. The acoustic effects as seen in the twin vocal folds, e.g., lower and loud calls, are often argued to have an evolutionary advantage for vocal exaggeration of body size in primates, e.g. the huge hyoids of howlers^12,17,37^. Thus, ancestors with VVFs may have had such a selective advantage compared with conspecifics without VVFs in Madagascar. Our multidisciplinary approaches indicated that similar acoustic effects may be achieved by evolutionary modifications in different features for separate clades, and that a unique evolutionary adaptation occurred in Madagascan lemurs.

Our analyses lack direct in-vivo evidence, e.g., from high-speed video recordings or electroglottography^6,7,11–13^, but here we provided anatomical and histological evidence to support the hypothesis that novel structures, VVFs, oscillate and collide in lemurs, as well as a mechanical model simulation.

## Materials and methods

### CT scanning

We used primate larynges preserved in 10% formalin at the Japan Monkey Centre (JMC; Supplementary Table 1), and no live animals or humans were involved in the study. Diffusible iodine-based contrast-enhanced computed tomography (DiceCT) was used to examine the anatomical specializations described in the study^6,38^. Before scanning, the specimens were soaked in 25%, 50% and 75% methanol for 10 min each in this order, and then in 100% methanol for 10 min three times for dehydration. Next, the dehydrated specimens were stained with iodine potassium iodide (I_2_KI)^6,38^. They were soaked in a 5% solution of I_2_KI for 1–30 days, depending on the condition of each specimen to stain. After these processes, the specimens were scanned with μCT scanner (Bruker SKYSCAN 1275) at the Center for the Evolutionary Origins of Human Behavior (EHUB) of Kyoto University. After scanning, these stained specimens were bleached using a 1% solution of sodium thiosulfate.

We examined the laryngeal anatomy using the μCT scans with Amira 3D (version 2021.1, Thermo Fisher Scientific). We used it to segment the area representing the laryngeal muscles and cartilages on each scan, manually supported by the Magic Wand tool, and to reconstruct the three-dimensional images.

### Histoanatomical analyses

We used the larynges preserved in 10% formalin at the JMC, the Fukuoka City Zoo (FCZ), and the Wildlife Research Center (WRC) of Kyoto University (Supplementary Table 1). They were extracted from primates that had died of natural causes, and no live animals or humans were involved in the study. The whole-organ serial section technique was employed^39^. The larynges were fixed in 10% formalin, dehydrated in graded ethanol concentrations, embedded in paraffin, and stained with hematoxylin and eosin (H & E). Transverse and coronal serial sections were made, and light microscopic observation was performed.

### Silicone compound model simulation

A physical model was constructed to study the oscillation properties of the twin vocal folds observed in the larynx of lemuriforms. The experiments on flow-induced oscillations of the physical model were conducted at the Ritsumeikan University.

**Figure 5:**
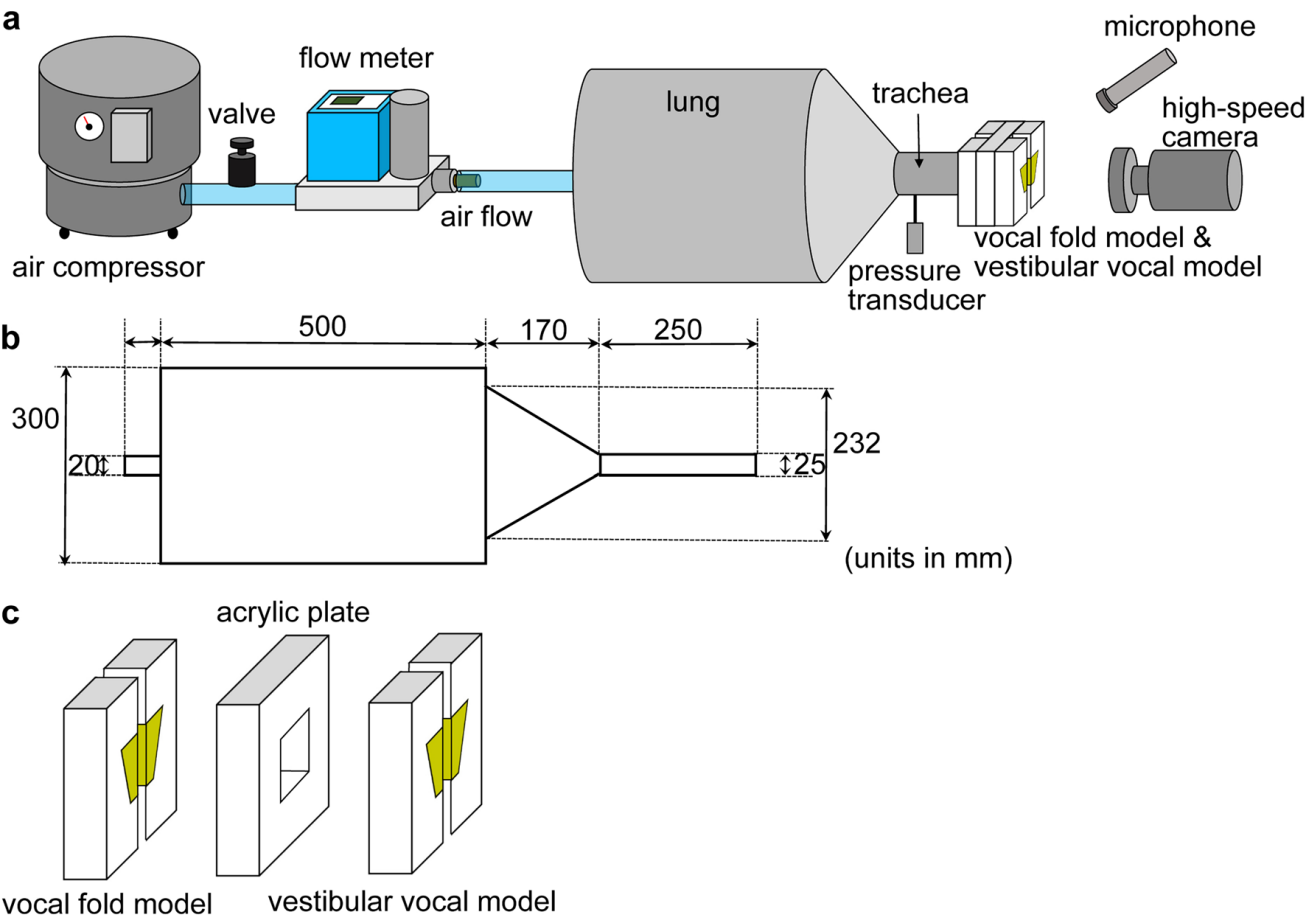
Schematic diagrams of the experimental equipment. a Experimental equipment. b Dimensions of the chamber and tracheal tube models. c Schematic illustration of the MRI models (vocal fold model and vestibular vocal fold model) and acrylic plate.

As a model for both the VFs and VVFs, the MRI model, a self-oscillating vocal fold model based on magnetic resonance imaging (MRI) data, was utilized^32^ (Fig. 4a). The model comprises two layers: the body and the cover. The surface is covered with a very soft superficial layer, enabling surface movement similar to the mucosal wave. The model was fabricated following the methodology of Murray and Thomson^32^ and Matsumoto et al.^33^. In the experiment, an airflow was injected through a pair of two MRI models (Fig. 5a, b). The upper and lower models represented the VVFs and the VFs for lemuriforms, respectively (Fig. 5c). Each model was attached to a 1.2-cm-thick rigid acrylic orifice plate (Fig. 5c). The distance between the VFs and VVFs in the inferior–superior direction was set to 10 mm. The medial–lateral distance between the left and right VVFs was changed from 0 to 10 mm by inserting a series of thin acrylic plates (each plate 0.5 mm thick) between them. Only the data with a distance of 1–6 mm were analysed since they exhibited co-oscillations of both the VFs and VVFs, while only the VFs vibrate outside of this range. The medial–lateral distance between the left and right VFs was always set to 0 mm.

As a trachea, a polyvinyl chloride tube (length: 25 cm, inner diameter: 2.5 cm) was connected to the VFs model (Fig. 5a, b). The airflow, which travels from an air pump (SilentAirCompressor Sc820, Hitachi Koki Co., Ltd.) to the tracheal tube, was controlled by a pressure regulator (10202U, Fairchild) and a digital mass flow controller (CMQ-V, Azbil, Fig. 5a). The subglottal pressure was measured by a pressure transducer (differential pressure transducer, DP15-28-N1S4A, Validyne Engineering; pressure amplifier PA501, KRONE Corporation), which was mounted flush in an inner wall of the tracheal tube, 2 cm upstream of the VFs model (Fig. 5a). The acoustic sound and the sound pressure level were measured by an omnidirectional microphone (Type 4192, Nexus conditioning amplifier, Brüel & Kjær) and a sound level meter (Type 2250-A, Brüel & Kjær), respectively, both located 10 cm from the model (Fig. 5a). A high-speed camera (FASTCAM NOVA S6, Photron) was used to observe oscillation patterns of the VFs and VVFs (representing the VFs and VVFs, respectively, Figs. 4b, 5a). From the high-speed video, which captures dynamics of the VFs and VVFs on medial–lateral and anterior–posterior axes, the medial–lateral movements were extracted into the kymogram by MATLAB software (R2021b, version 9.11.0, The MathWorks)^31^. All signals were stored in a digital recorder (controller, PXIe-8840; input/output card, BNC-2110; software, Labview, National Instruments) with a sampling frequency of 12.5 kHz.

To measure the phonation onset pressure, the flow rate was slowly increased from 0 l/min to a maximum value in 5 s. Since the flow rate needed to induce oscillations of the VFs and VVFs depended upon the individual experimental setting, its maximum value was adjusted in a range from 0.5 to 3.5 l/min. The phonation onset was detected at the pressure, where the difference between the maximum and minimum subglottal pressure exceeded a threshold value. After the onset point, self-sustained oscillations of the VFs model were continued to be measured by the microphone and the sound level meter, from which the fundamental frequency and the vocal efficiency were computed by the MATLAB software and the Praat software (www.praat.org, Version 6.1.53). For each pair of the VFs and VVFs, the fundamental frequency and onset pressure were measured two or three times. The experiment was repeated for five pairs of the VFs and VVFs models. To clarify the effect of the VVFs, the same experiment was conducted for only oscillation of the VFs model without oscillation of the VVFs model.

### Two-way ANOVA

The average was taken from the data of the fundamental frequency, vocal efficiency, phonation onset pressure which were obtained from two or three repetitions under each condition. Then, the average of the data for the same conditions of width and presence/absence of VVFs model oscillation was taken. Next, a two-way ANOVA was performed using RStudio (version 1.4.1717, PBC). Two-way ANOVA was used to test whether the effects of the two factors on the distance conditions and the presence/absence of VVFs vibration were each recognized and whether there was an interaction between the two factors. Each distance condition is regarded as a discrete variable. Based on the obtained data, graphs for each parameter were drawn using RStudio.

### Supplementary Information

Supplementary Information.

## Data Availability

All data are available in the main text or the supplementary materials.
