# Supplementary material for: Twin vocal folds as a novel evolutionary adaptation for vocal communications in lemurs
Source: Sci Rep. 2024 Feb 13;14:3631. doi: 10.1038/s41598-024-54172-z (PMC10864409; doi:10.1038/s41598-024-54172-z)
Supplement: Supplementary file 1 — Supplementary Information. [file 41598_2024_54172_MOESM1_ESM.docx]

**Supplementary Figures and Table**


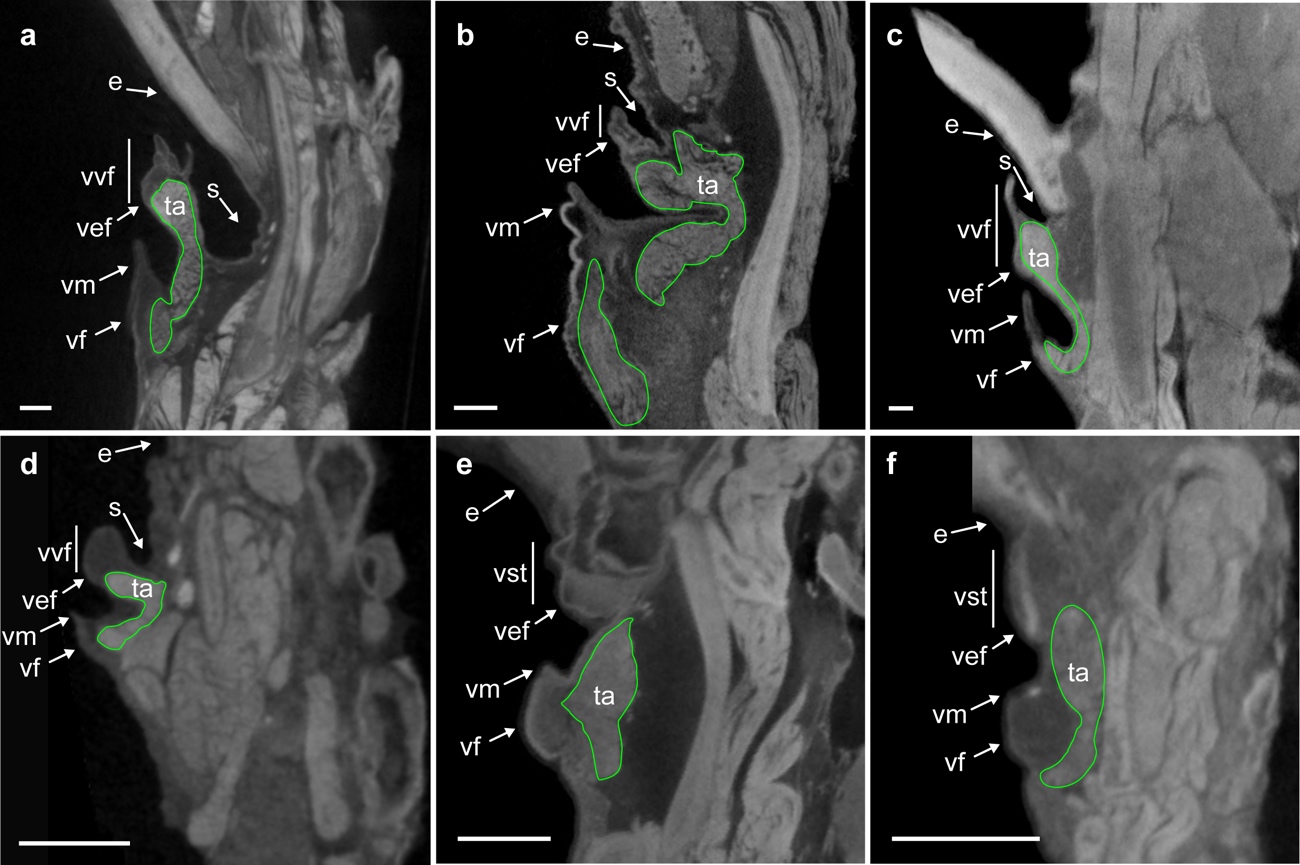


**Supplementary Figure 1** **CT frontal scans of the half larynx.** Lemuriform: (**a**) *Eulemur macaco,* (**b**) *Eulemur mongoz*, (**c**) *Varecia variegata*, and (**d**) *Microcebus murinus*. Lorisiform: (**e**) *Otolemur crassicaudatus*, and (**f**) *Galago senegalensis*. e, epiglottis; s, sulcus; ta (segmented in green), thyro-arytenoid (TA) muscle; vef, ventricular fold; vf, vocal fold; vm, vocal membrane; vst, vestibule; and vvf, vestibular vocal fold. Scale, 1 mm.


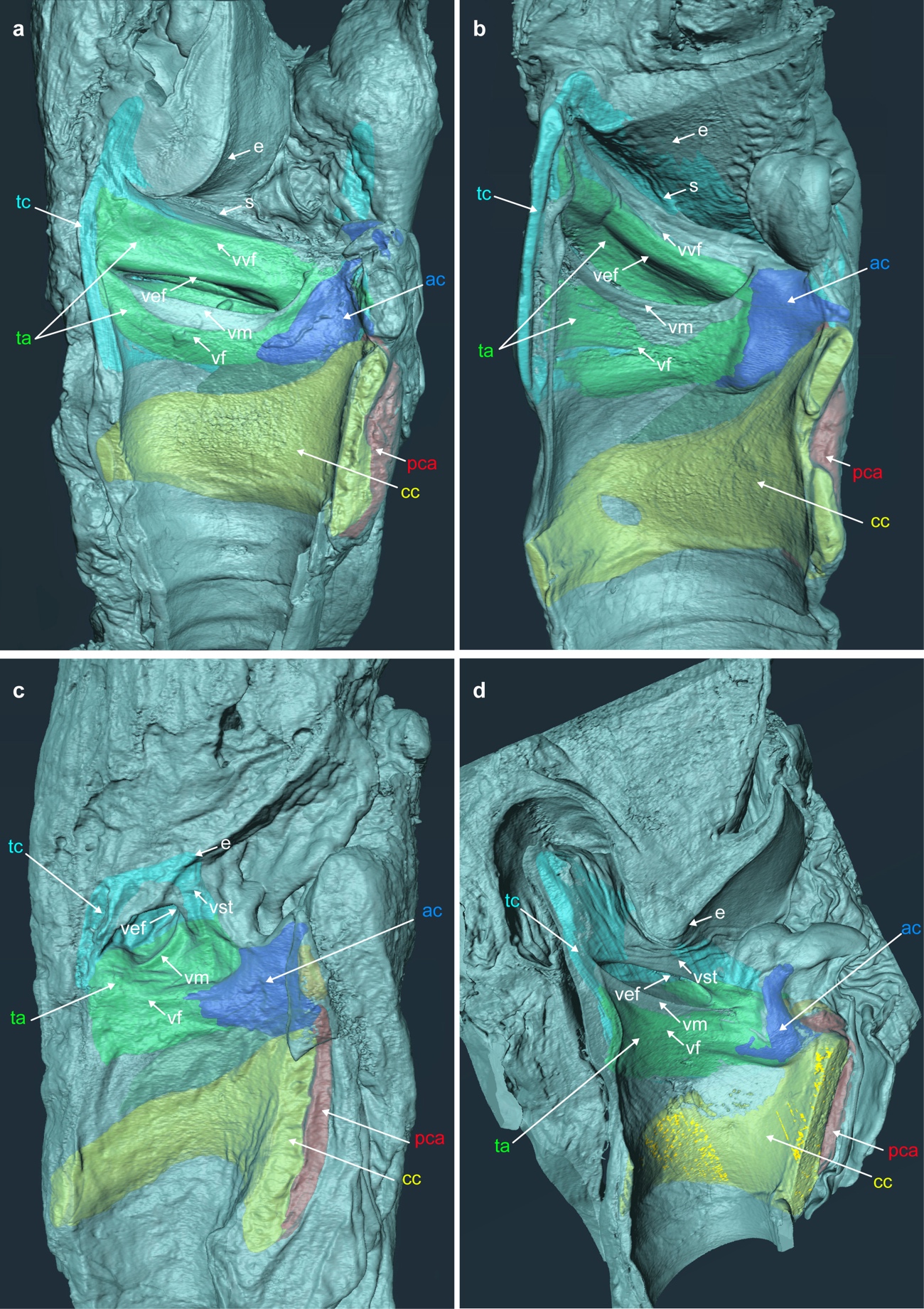


**Supplementary Figure 2.** **3D images of the larynx.** Lemuriform: (**a**) *Lemur catta* and (**b**) *Eulemur macaco*; lorisiform: (**c**) *Nycticebus coucang*; and anthropoid: (**d**) *Macaca fuscata*. ac (dark blue), arytenoid cartilage; cc (yellow), cricoid cartilage; e, epiglottis; pca (red), posterior cricoarytenoid muscle; s, sulcus; ta (green), thyro-arytenoid (TA) muscle; tc (light blue), thyroid cartilage; vef, ventricular fold; vf, vocal fold; vm, vocal membrane; vst, vestibule; and vvf, vestibular vocal fold.


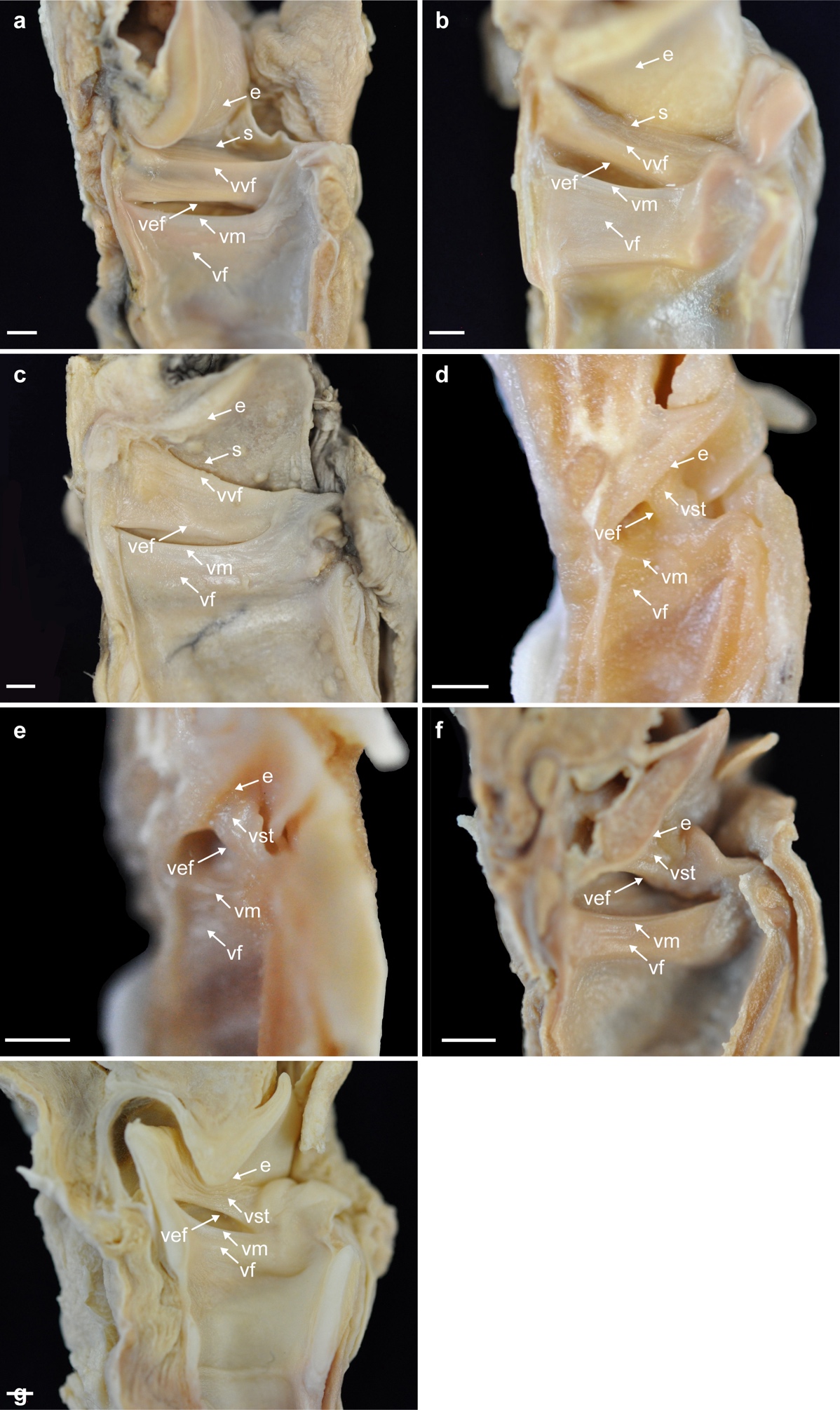


**Supplementary Figure 3.** **Medial views of larynges.** Lemuriform: (**a**) *Lemur catta*, (**b**) *Eulemur macaco*, and (**c**) *Varecia variegata*; lorisiform: (**d**) *Nycticebus coucang*, (**e**) dorsal view of *Nycticebus coucang*, and (**f**) *Otolemur crassicaudatus*; and anthropoid (**g**) *Macaca fuscata*. e, epiglottis; s, sulcus; vef, ventricular fold; vf, vocal fold; vm, vocal membrane; vst, vestibule; and vvf, vestibular vocal fold. Scale, 2 mm.

**
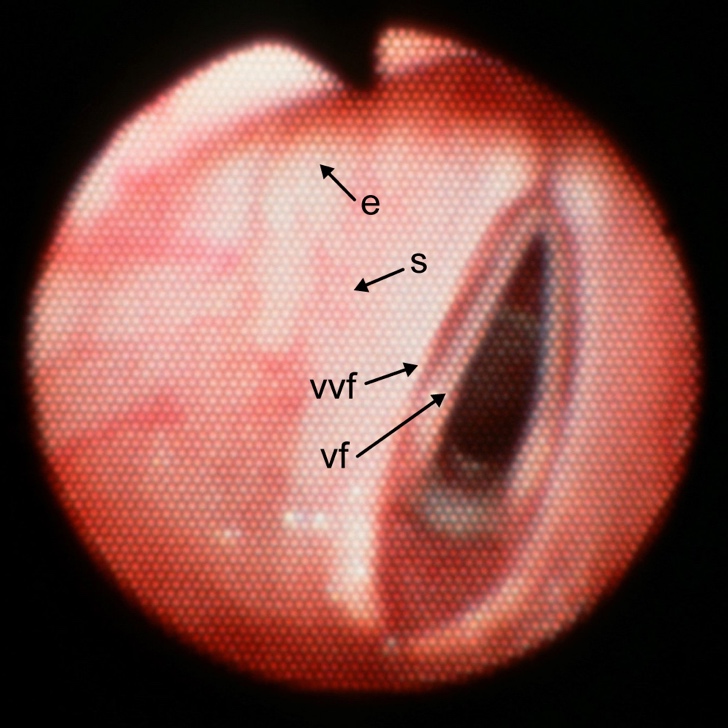
**

**Supplementary Figure 4. A laryngeal endoscopic image of lemuriform *Lemur catta*.** e, epiglottis; s, sulcus; vf, vocal fold; and vvf, vestibular vocal fold.


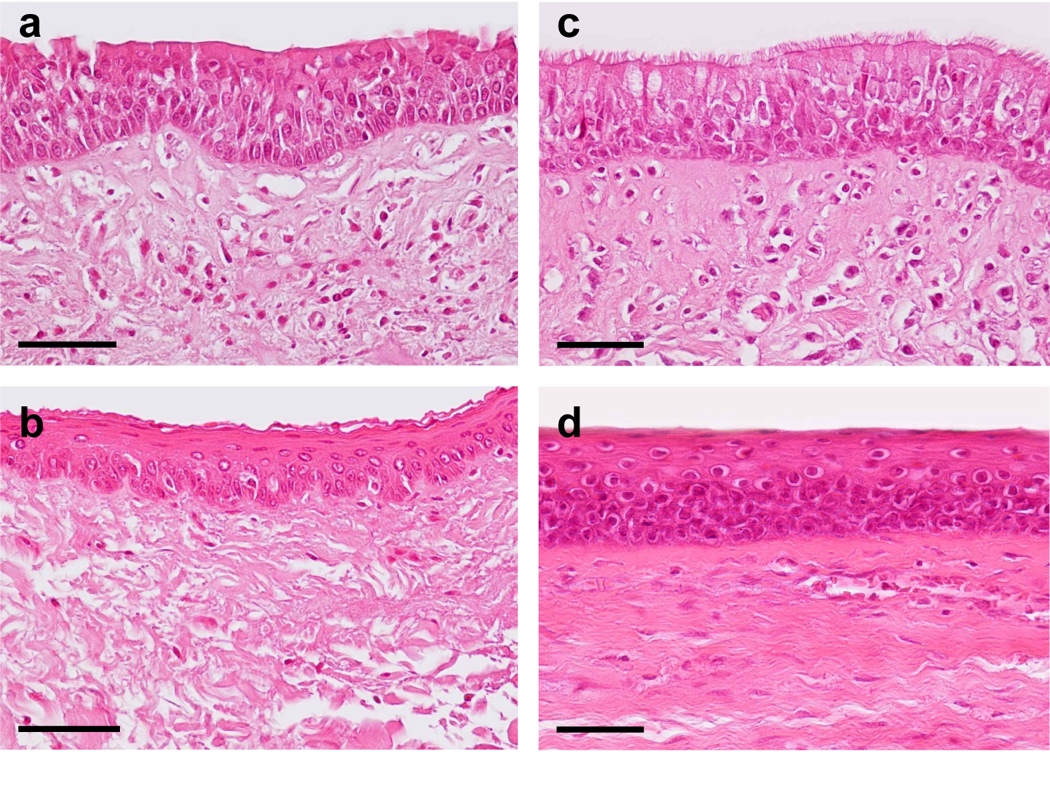


**Supplementary Figure 5. H & E stained histo-anatomical sections in anthropoids (hominids).** **a** Vestibule and **b** vocal fold of *Pan troglodytes*, and **c** vestibule and **d** vocal fold of *Gorilla gorilla*. Scale 50 μm.

**Supplementary Table 1.** **Specimens used in this study.**

| Species | ID/name of specimens | |
| --- | --- | --- |
|  | CT scan | Histological section |
| Lemuriformes |  |  |
| Lemuridae |  |  |
| *Lemur catta* | Pr6248 (M) | Pr6248 (M) |
| *Eulemur macaco* | Pr6526 (F) |  |
| *Eulemur mongoz* | Pr2559 (F) |  |
| *Varecia variegata* | Pr6335 (M) |  |
| Cheirogaleidae |  |  |
| *Microcebus murinus* | Pr5316 (M) |  |
| Lorisiformes |  |  |
| Lorisidae |  |  |
| *Nycticebus coucang* | Pr3365 (F) |  |
| Galagidae |  |  |
| *Otolemur crassicaudatus* | Pr4308 (M) |  |
| *Galago senegalensis* | Pr6341 (F) |  |
| Catarrhini |  |  |
| Cercopithecidae |  |  |
| *Macaca fuscata* | Pr6493 (F) | Pr6718 (F) |
| Hominidae |  |  |
| *Gorilla gorilla gorilla* |  | Willie (M) |
| *Pan troglodytes verus* |  | Sanzou (M) |

M, male; and F, female
